# Gastrulation EMT Is Independent of P-Cadherin Downregulation

**DOI:** 10.1371/journal.pone.0153591

**Published:** 2016-04-20

**Authors:** Pricila K. Moly, James R. Cooley, Sebastian L. Zeltzer, Tatiana A. Yatskievych, Parker B. Antin

**Affiliations:** Department of Cellular and Molecular Medicine, University of Arizona, 1656 E. Mabel Street, P.O. Box 245217, Tucson, AZ, 85724, United States of America; Texas A&M University, UNITED STATES

## Abstract

Epithelial-mesenchymal transition (EMT) is an evolutionarily conserved process during which cells lose epithelial characteristics and gain a migratory phenotype. Although downregulation of epithelial cadherins by Snail and other transcriptional repressors is generally considered a prerequisite for EMT, recent studies have challenged this view. Here we investigate the relationship between E-cadherin and P-cadherin expression and localization, Snail function and EMT during gastrulation in chicken embryos. Expression analyses show that while E-cadherin transcripts are detected in the epiblast but not in the primitive streak or mesoderm, P-cadherin mRNA and protein are present in the epiblast, primitive and mesoderm. Antibodies that specifically recognize E-cadherin are not presently available. During EMT, P-cadherin relocalizes from the lateral surfaces of epithelial epiblast cells to a circumferential distribution in emerging mesodermal cells. Cells electroporated with an E-cadherin expression construct undergo EMT and migrate into the mesoderm. An examination of Snail function showed that reduction of Slug (SNAI2) protein levels using a morpholino fails to inhibit EMT, and expression of human or chicken Snail in epiblast cells fails to induce EMT. In contrast, cells expressing the Rho inhibitor peptide C3 rapidly exit the epiblast without activating Slug or the mesoderm marker N-cadherin. Together, these experiments show that epiblast cells undergo EMT while retaining P-cadherin, and raise questions about the mechanisms of EMT regulation during avian gastrulation.

## Introduction

During gastrulation in amniote organisms, epiblast cells undergo an epithelial-to-mesenchymal transition (EMT) in the primitive streak to form the mesoderm and endoderm cell layers. EMT is driven by large-scale changes in gene expression involving the downregulation of the epithelial phenotype, including apical basal polarity, in epiblast cells and upregulation of genes that confer migratory behavior, and front-back polarity, to the emerging mesoderm [1, 2]. Cells contributing to the endoderm reestablish junctional complexes, while mesoderm cells remain migratory for variable lengths of time before reestablishing epithelial layers during the early stages of organ formation. During embryogenesis, cells can progress through repeated cycles of EMT and the reverse process mesenchymal-epithelial transition (MET; [3]).

Although diverse signaling pathways can regulate EMT depending on the cellular context [4], activation of transcriptional repressors of the Snail, Twist and Zeb families is a broadly conserved early step [5]. During EMT, Snail zinc finger proteins (Snail and Slug, transcribed from the SNAI1 and SNAI2 genes, respectively) directly repress transcription of genes involved in the epithelial phenotype, including components of the tight junction and adhesion proteins such as E-cadherin (E-cad; [6–10]). The cadherins are transmembrane proteins that mediate intercellular adhesion through homophilic and heterophilic interactions between their extracellular cadherin domains [11]. Cadherin intracellular domains link to the cytoskeleton through a protein scaffold that includes α-catenin, β-catenin and p120-catenin. During gastrulation, published studies have reported that E-cad expressed in the epiblast is replaced by N-cadherin (N-cad) in the mesoderm.

Although E-cad downregulation and the accompanying reduction in adhesion have often been considered a prerequisite for EMT [12–14], recent studies have challenged this view. For example, while *Drosophila* embryos lacking Snail fail to form a ventral furrow and are deficient in mesoderm [15], neither ablation nor over expression of E-cad (*shg*) or N-cad (*CadN*, *CadN2*), or both, has any effect on formation of the ventral furrow, germ layer formation or mesoderm spreading [16]. Similarly, although E-cad is required for epithelial architecture and branching morphogenesis in mammary epithelium, loss of E-cad does not lead to cell dissemination [17]. In fact the opposite appears to be true since Twist-1 induced cell dissemination of mammary epithelial cells requires E-cad.

Protein localization studies in gastrulating chicken embryos also suggest that downregulation of epithelial cadherins is not a prerequisite for EMT. Although E-cad mRNAs expressed in the epiblast are lost as cells approach the primitive streak, immunofluorescence analyses using antibodies recognizing the intracellular domain of E-cad have reported robust protein levels on the surface of cells as they undergo EMT [1, 2]. However, interpretation of these findings is complicated by the co expression of P-cadherin (P-cad), a closely related protein transcribed from the *CDH3* gene that is adjacent to the gene coding for E-cad (*CDH1*). P-cad and E-cad share identical cytoplasmic domains, and so both are recognized by the commercially available E-cad antibodies used in published studies. The high sequence similarity between E-cad and P-cad suggest some functional redundancy, though this has not been experimentally tested.

Here we investigate the relationship between E-cad and P-cad expression, Snail function and EMT during gastrulation in chicken embryos. We find that P-cad is retained on the surface of cells undergoing EMT in the primitive streak and after they emerge in the mesoderm, while evidence suggests that E-cad is downregulated prior to the onset of EMT. Overexpression of E-cad in chicken embryos did not alter the ability of epiblast cells to undergo EMT. Furthermore, gain and loss of function studies suggest that Slug (SNAI2) is neither required for gastrulation EMT nor sufficient to induce EMT in the epiblast.

## Materials and Methods

### Embryo collection

Fertile chicken (*Gallus gallus*) eggs (Hy-Line Intl., Iowa; not a commercially available source) were incubated at 37°C with high humidity until embryos reached Hamburger-Hamilton (HH) stage 3 to 4 [18, 19]. WT C57Bl6 mouse embryos were dissected from females at 7.5 dpc. All of the animals handling protocols and procedures specific to this manuscript were approved by the IACUC of the University of Arizona. Chicken and mouse embryos were washed in PBST, fixed for 2 hours in freshly prepared 4% paraformaldehyde at room temperature (R.T.), then dehydrated through a graded MEOH series and stored at -20°C overnight in 100% MEOH.

### Antibodies

The following primary antibodies were used for immunofluorescence: rabbit anti-GFP (1:500, Invitrogen); rabbit anti-FLAG (1:500, Cell Signaling); mouse anti-E-cadherin (1:500, BD Biosciences); mouse anti-N-cadherin (1:250, Sigma-Aldrich); rabbit anti-laminin (1:500, Abcam); rabbit monoclonal anti-Slug (1:400, Cell Signaling); rabbit ZO1 (1:100, Santa Cruz Biotechnology); rabbit anti-Myc monoclonal (1:300, Cell Signaling); mouse anti-c-Myc (1:300, Santa Cruz Biotechnology); mouse anti-GM130 (1:50, BD Biosciences); mouse anti-fluorescein (1:250, Roche); and anti-p120-catenin (1:50, Santa Cruz Biotechnology). The E-cadherin antibody was prepared against the intracellular domain that is 100% identical to the intracellular domain of P-cadherin, and so recognizes both proteins. An E-cadherin-specific antibody could not be identified, and attempts to produce one were not successful (see below). The following secondary antibodies were obtained from Invitrogen and used at 1:500 dilution: Alexa fluor 488 conjugated goat anti-rabbit; Alexa fluor 488 conjugated goat anti-mouse IgG1; Alexa fluor 488 conjugated goat anti-mouse IgG2a; Alexa fluor 594 conjugated goat anti-rabbit; Alexa fluor 594 conjugated goat anti-mouse IgG1; Alexa fluor 594 conjugated goat anti-mouse IgG2a; Alexa fluor 647 conjugated goat anti-rabbit; Alexa fluor 647 conjugated goat anti-mouse IgG1; Alexa fluor 647 conjugated goat anti-mouse IgG2a.

For the detection of P-cadherin protein, an affinity-purified antibody was generated in rabbit using the peptide GRVESCAQKPRVDTGVP corresponding to amino acids 541–557 of the extracellular domain that shows only 29% identity to the corresponding sequence in E-cad. E-cadherin-specific antibodies were prepared using the peptides LKKELEPGEYNIFVKL and YETKSRYDLVVTVENKV, corresponding to amino acids 659 to 673 and 450 to 467, respectively, of the extracellular domain. Although the resulting affinity purified antibodies recognized the corresponding E-cad antigen peptides, attempts to use these antibodies to detect E-cad protein by immunocytochemistry were unsuccessful.

### Electroporation, Immunohistochemistry, In situ Hybridization and Cell Analyses

Electroporation and subsequent immunohistochemistry were carried out essentially as described [2, 20] using an Intracel TSS20 Ovodyne electroporator at the following settings: three 500ms pulses at 4V spaced 1s apart. HH stage 2–4 embryos were placed in New culture [21] and electroporations performed using conditions in which only epiblast cells were electroporated [20]. The following plasmids were electroporated at a concentration of 1-2ug/ml: pBE-eGFP; pBE-6SAhSNAI1, expressing a FLAG-tagged dominant active form of human Snail; pdeltaZf-Snail2, expressing a Myc-tagged dominant negative chicken Snail2 (Slug) lacking the transactivation domain [22]; pC3, expressing the C3 exoenzyme, a potent Rho pathway inhibitor [23]; pECad, expressing FLAG-tagged full length chicken E-cadherin. For morpholino knockdown studies, a fluorescein-labeled morpholino (TGGCATTTTGAAGGCAGGCTTTCTC) targeting the initiation of translation of the *SNAI2* mRNA, or a five base pair mismatch control morpholino (GeneTools), were electroporated at a concentration of 0.5uM along with 0.5ug/ml of carrier plasmid. Following electroporation, embryos were incubated for 3–16 hours before fixation and processing for immunohistochemistry and/or in situ hybridization. For immunofluorescence, embryos were fixed for 2 hours at R.T. in freshly prepared 4% paraformaldehyde, dehydrated through methanol and stored at -20°C overnight. After rehydration, embryos were blocked in 5% goat serum in PBST for 1 hour at room temperature before incubation overnight at 4°C with primary antibody diluted in blocking solution. Embryos were then washed extensively and incubated overnight at 4°C with the appropriate secondary antibody conjugated to Alexa fluor 488, Alexa fluor 594 or Alexa fluor 647. For In situ Hybridization (ISH), embryo processing, antisense RNA probe preparation and whole-mount ISHs were performed as described [2]. For embryos processed for combined immunofluorescence and ISH, immunofluorescence was performed after ISH. Following extensive washing, embryos were imaged in whole mount on a Leica MZ16FA stereomicroscope and then processed into Paraplast for sectioning at 8–12 μm. Transverse section images were captured on a Zeiss Meta510 confocal microscope.

Cell localization in the epiblast, primitive streak, and mesoderm was analyzed as previously described [20]. Significant differences were calculated with the Student's T-test feature of Microsoft Excel. Standard deviations were calculated in Microsoft Excel.

## Results

### Gene expression during EMT

**Fig 1:**
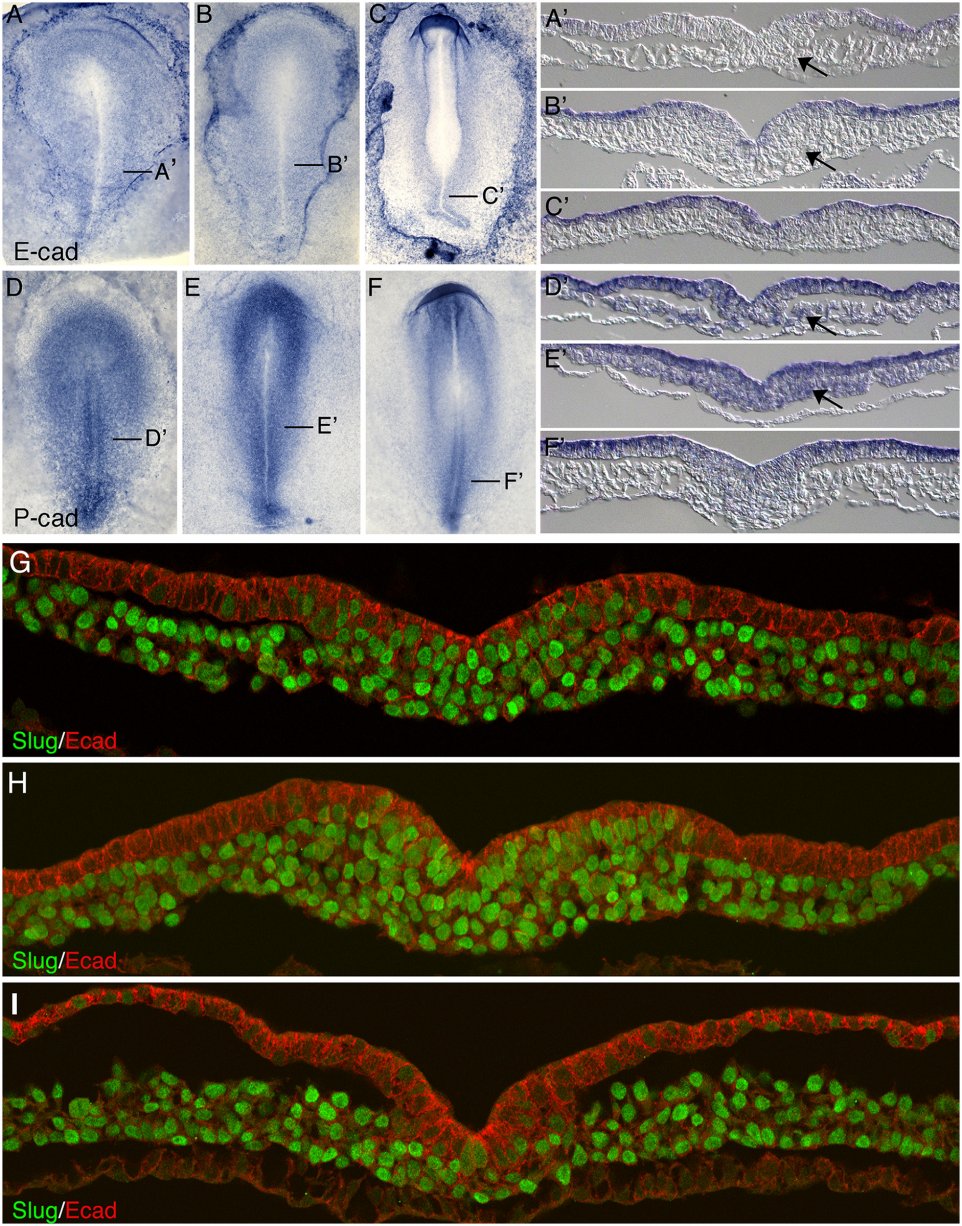
Localization of E-cad and P-cad mRNA, and E-cad and Slug proteins, in HH stage 4–7 embryos. (A-C, A’-C’) E-cad (*CDH1*) mRNA expression in HH stage 4, 5 and 7 embryos (A-C) and in transverse sections (A’-C’). (D-F, D’-F’) P-Cad (*CDH3*) mRNA expression in embryos at stages 4, 5 and 6 (D-F) and in transverse sections (D’-F’). E-cad mRNAs are downregulated in the pre ingression epiblast and in the primitive streak, while P-cad mRNAs persist in the primitive streak and medial mesoderm (arrows). (G-I) Immunofluorescence localization of Slug (green) and cadherin proteins using an antibody that recognizes E-cad and P-cad (red) in transverse sections through the middle streak region of HH stage 4, 5 and 7 embryos.

The movement of cells from the epiblast to the mesoderm via an EMT has been correlated with the loss of E-cad (*CDH1*) and the appearance of N-cad (*CDH2*) [1–3]. In chicken embryos, E-cad transcripts are expressed throughout the epiblast but are absent from the primitive streak (Fig 1A–1C and 1A’–1C’). The loss of E-cad mRNAs coincides with Slug protein expression (*SNAI2*; Fig 1G–1I). P-cadherin (P-cad) is encoded by the *CDH*3 gene, which is adjacent to and shows high sequence similarity to *CDH1*. The intracellular domains of P-cad and E-cad are identical, while the extracellular domains have diverged. P-cad transcripts are also detected in the epiblast, but additionally are evident in the primitive streak and in newly formed mesoderm cells (Fig 1D–1F and 1D’–1F’).

All available E-cad antibodies have been prepared using sequences from the cytoplasmic domain as immunogens, and so recognize both E-cad and P-cad. To examine the relative localization of P-cad and E-cad, antibodies were generated against extracellular domain peptides that showed low identity to the corresponding peptide in the other protein. While a P-cad specific antibody was obtained that is useful for immunocytochemistry, we were unable to generate an E-cad specific antibody that could visualize the protein in cells.

**Fig 2:**
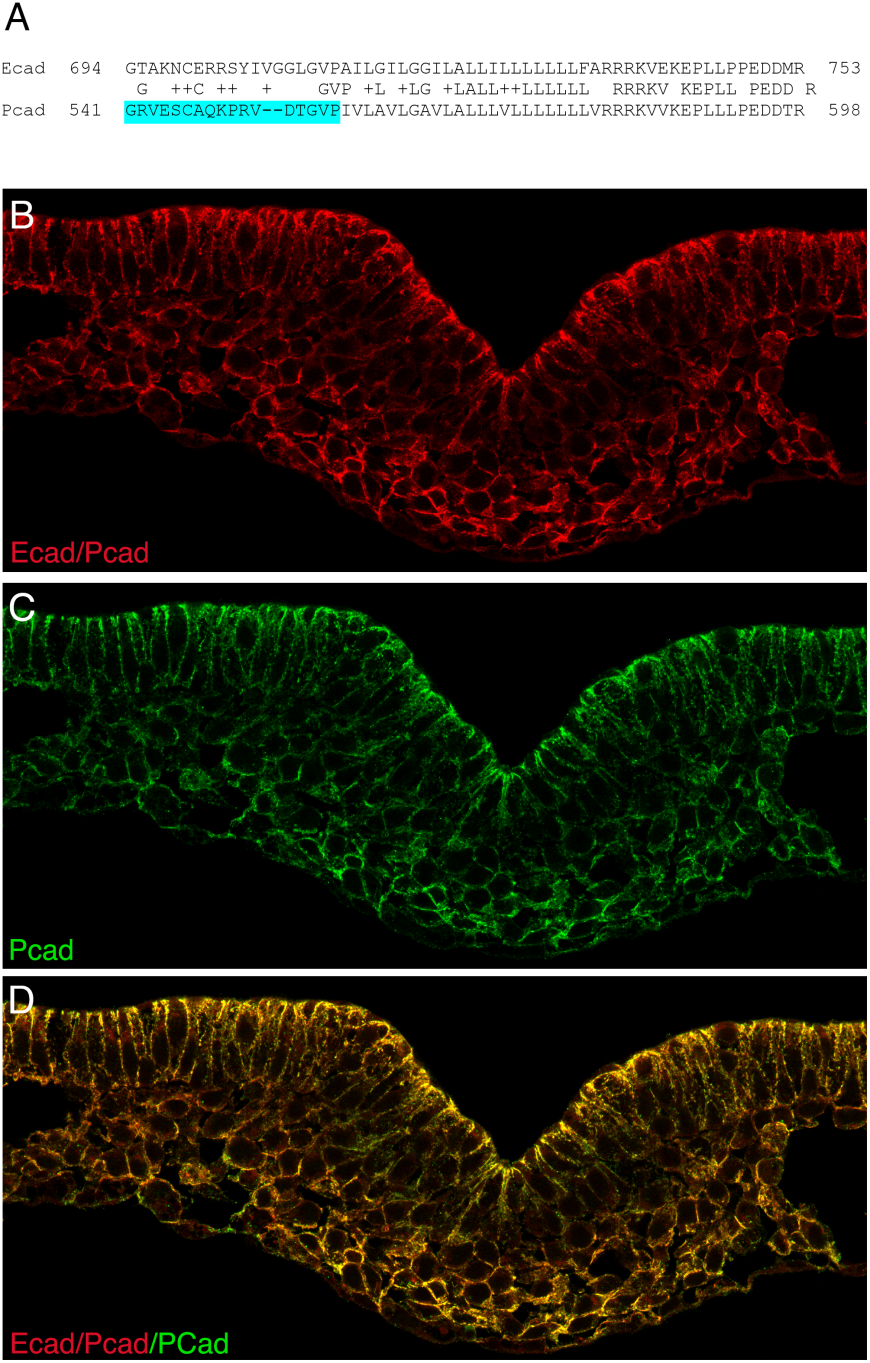
Co localization of E-cad and P-cad in using a P-cad-specific antibody prepared against a peptide from the extracellular domain of P-cad that shares only three amino acids with the corresponding sequence in E-cad (A), and a commercially obtained antibody raised against the E-cad intracellular domain that recognizes both P-cad and E-cad. (B-D) Transverse section through the mid streak region of a HH stage 4 embryos, showing immunolocalization E-cad and/or P-cad (B), or P-cad (C), or both (D).

**Fig 3:**
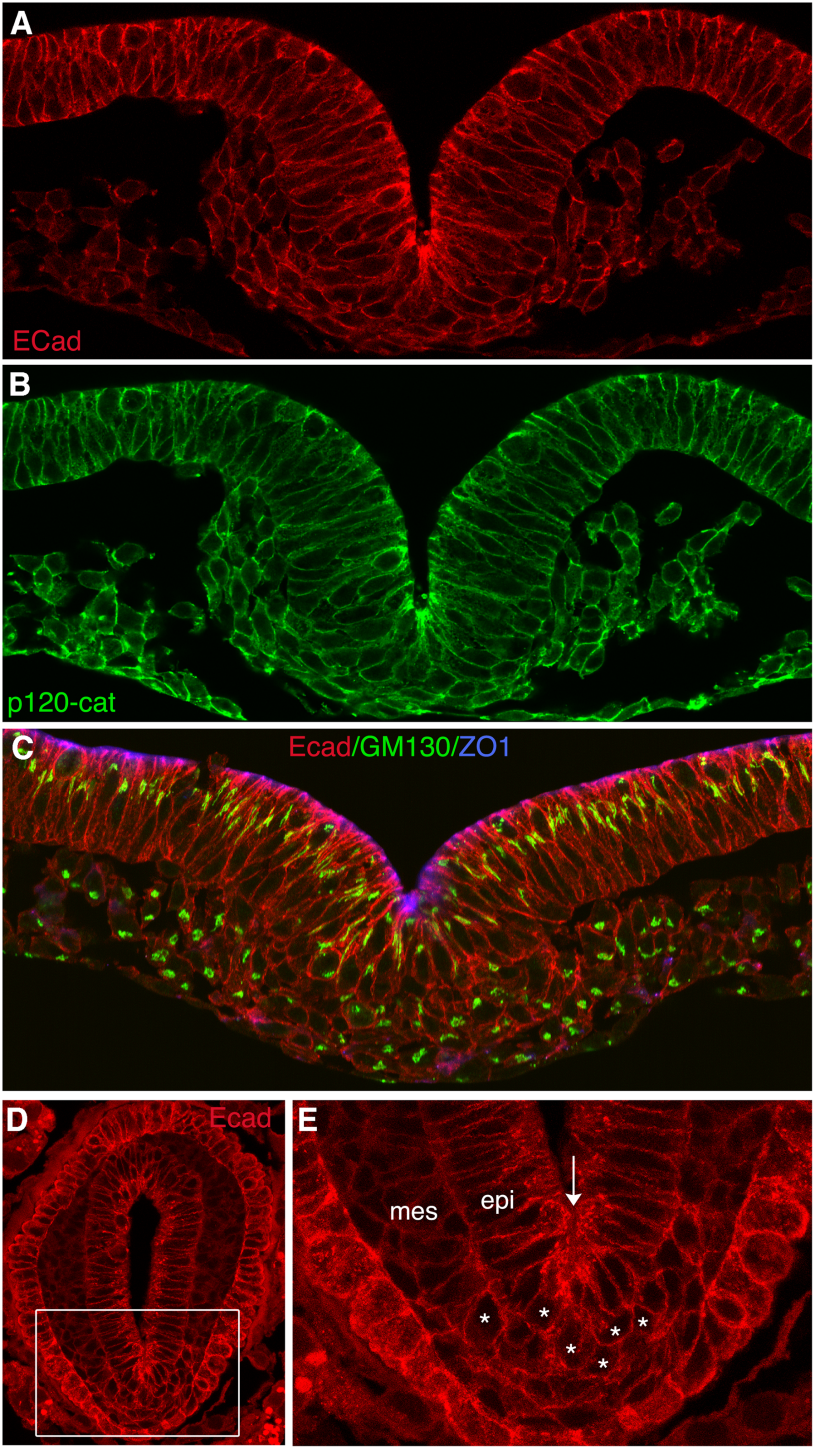
Protein localization in transverse sections of gastrulating chicken and mouse embryos. (A-C) Localization of E-cad and/or P-cad, p120-catenin, GM130 and ZO1 in transverse sections of HH stage 4 embryos. (A, B) The same microscopic field showing expression of E-cad and/or P-cad (A) and p120-catenin (B). The proteins colocalize at the periphery of cells in the epiblast, primitive streak and mesoderm. (C) Colocalization of E-cad and/or P-cad, GM130 and ZO1in a transverse section of a HH stage 4 embryo. Cells moving from the epiblast to the mesoderm retain E-cad and/or P-cad at their periphery while exhibiting a reorientation of Golgi that is characteristic of the change in cell polarity associated with EMT. (D) Transverse section through a E7.5 mouse embryo at the level of the primitive streak, visualizing E-cad and/or P-cad proteins. (E) Higher magnification of the boxed area in (D), showing persistence of E-cad and/or P-cad proteins on the surface of cells in the mesoderm below and near the primitive streak (arrow). Asterisks are rounded cells in the mesoderm that retain E-cad and/or P-cad protein.

Immunolocalization of cadherin proteins using the P-cad specific antibody and an intracellular domain antibody that recognizes P-cad and E-cad is shown in Fig 2. P-cad protein is present along the lateral domains of all cells in the epiblast, primitive streak and newly formed mesoderm. The extent of protein expression corresponds to the expression domain of P-cad mRNA (Fig 1). Although we cannot definitively localize E-cad protein, E-cad mRNAs are present in epiblast cells but are not detectable in the primitive streak or mesoderm. Assuming that the expression domain of E-cad protein corresponds generally to its mRNA expression domain, E-cad protein would be diminished prior to the onset of EMT while P-cad protein is present at the periphery of cells in the primitive streak and mesoderm. Cells that have undergone EMT can be identified by a change in shape from elongated to round, by the re-distribution of P-cad from lateral-restricted in the epiblast to circumferential in the mesoderm, and by reorganization of the golgi from highly elongated in the epiblast to globular in cells of the ventral streak and mesoderm (Fig 3C; [1]).

**Fig 4:**
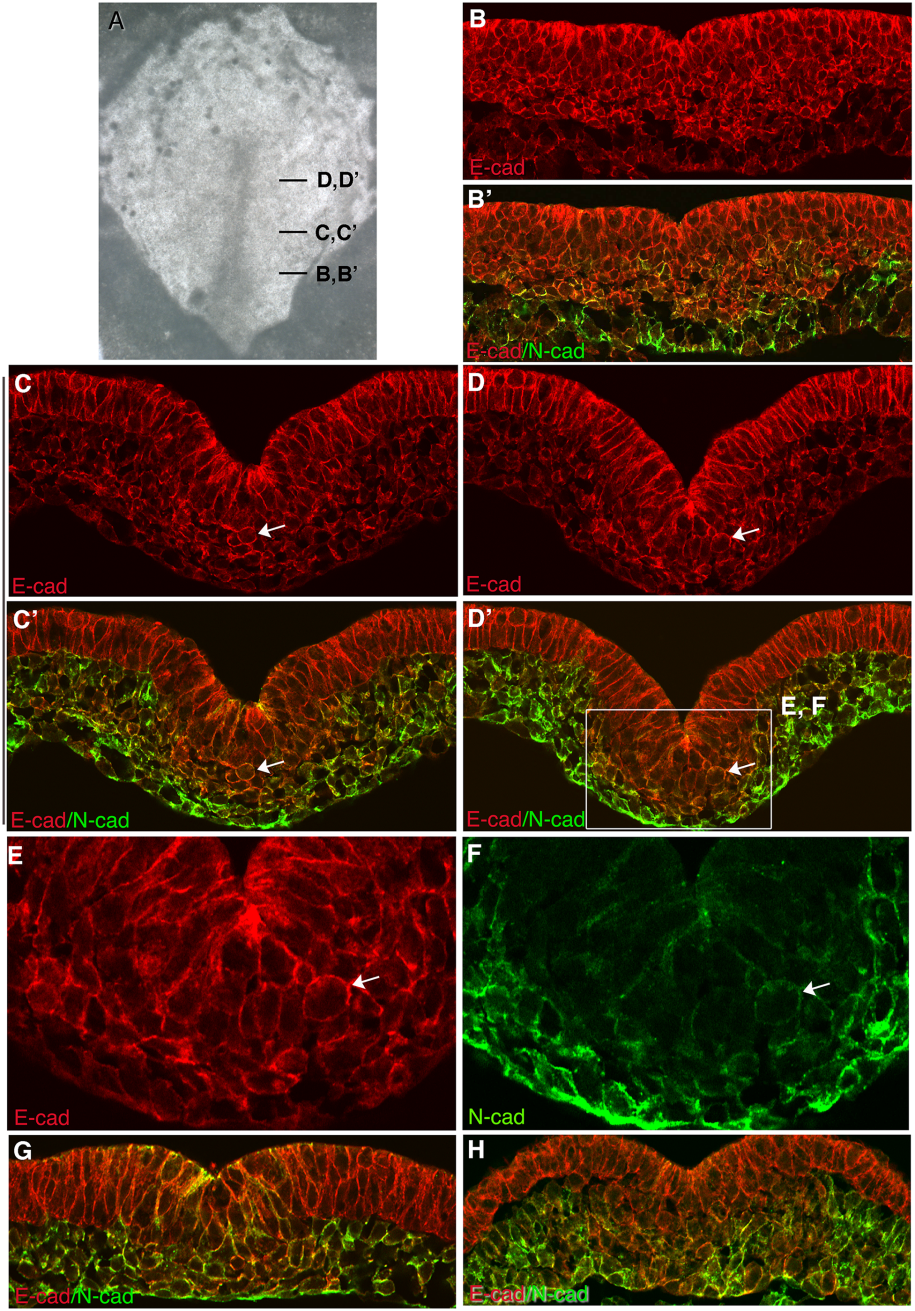
Localization of E-cad and/or P-cad and N-cad in transverse sections of a HH stage 3–4 chicken embryos. (A) Brightfield image of a HH stage 3 embryo following processing for immunofluorescence localization of E-cad and/or P-cad and N-cad, showing the location of images in (B-D’). Expression of E-cad and/or P-cad (B,C,D), or E-cad and/or P-cad plus N-cad (B’, C’, D’) in transverse sections. (E, F) Higher magnification images of boxed area in (D’), showing the localization of E-cad and/or P-cad (E) and N-cad (F) as cells transition from epiblast to mesoderm. E-cad and/or P-cad are retained on the surface of cells during EMT and after cells emerge into the mesoderm. Arrows point to rounded cells in the ventral streak showing robust detection of E-cad and/or P-cad at the cell periphery. (G, H) Co localization of E-cad and/or P-cad and N-cad in transverse sections of HH stage 4 embryos, showing their heterogeneous expression between mesoderm cells.

An antibody specific for N-cad was used to determine the relative localization of E-cad/P-cad (using the intracellular domain antibody that recognizes both proteins) and N-cad. N-cad is detected weakly in cells of the dorsal and mid streak and more robustly in mesoderm cells away from the primitive streak (Fig 4B’, 4C’, 4D’ and 4F–4H). The relative levels of E-cad/P-cad versus N-cad vary between individual cells in the primitive streak and mesoderm (Fig 4C’, 4D’ and 4E–4H). Similar heterogeneity has been observed in a number of endoderm-derived epithelial cells [24]. In all locations, E-cad/P-cad and N-cad predominantly co-localize to the cell periphery along with other intracellular cadherin complex proteins such as p120-catenin (Fig 3A and 3B). A similar pattern of cadherin localization was observed during gastrulation in mouse embryos (Fig 3D and 3E), where high levels of E-cad/P-cad proteins were detected on the surface of cells in the epiblast, in cells undergoing EMT during gastrulation, and in newly emerged mesoderm cells.

**Fig 5:**
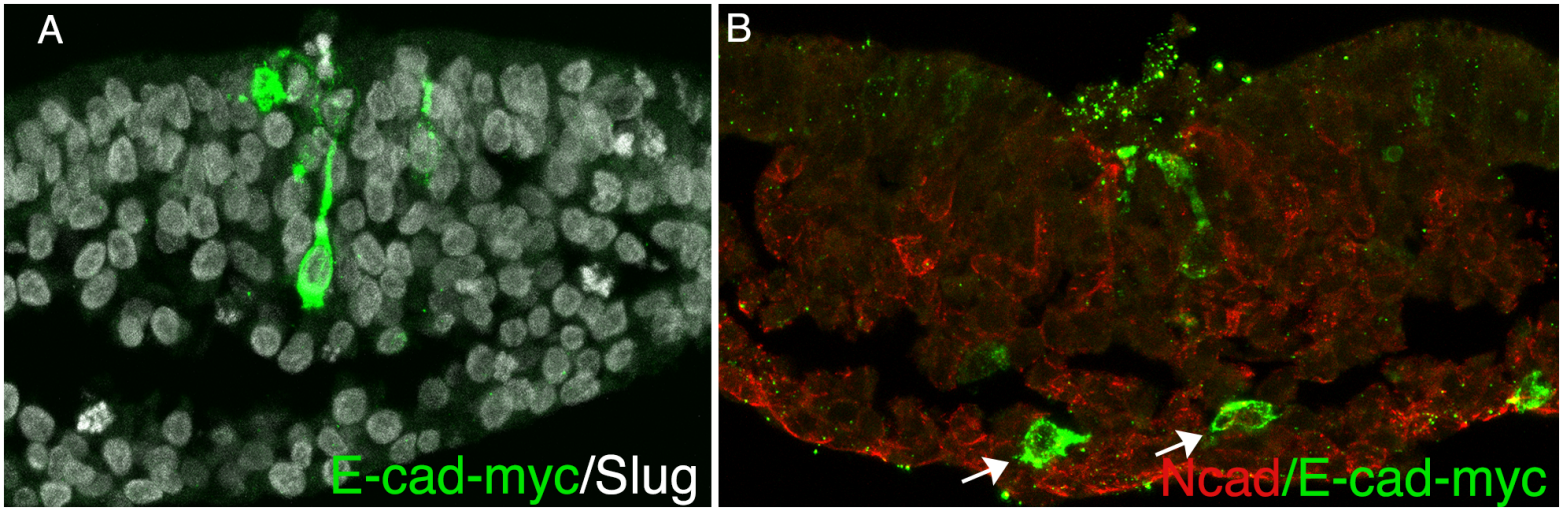
E-cad overexpression in gastrulating cells. (A-B) Transverse sections of the primitive streak region of embryos electroporated with a myc tagged E-cad expression construct, visualizing in (A) myc (green) and Slug (white), and in (B) myc (green) and N-cad (red). Expression of E-cad had no effect on the ability of cells to undergo EMT and migrate into the mesoderm (arrows in B).

To determine whether over expression of E-cad could inhibit EMT, the epiblast was electroporated with an E-cad expression construct. Cells expressing high levels of E-cad were observed undergoing EMT and residing in the mesoderm (Fig 5).

### Reduction of Slug expression fails to block migration of epiblast cells into the mesoderm

It is well established that the Snail family of transcriptional repressors downregulate E-cad as well as other genes associated with the epithelial phenotype [3, 6]. Slug protein (*SNAI2*) is present in the nuclei of cells in the primitive streak and the mesoderm of avian embryos (Fig 1G–1I; [25]), and gene ablation or inhibition of Slug function have been reported to impede cell movements during gastrulation [25, 26]. To explore in more detail the relationship between Slug function and EMT during gastrulation, a fluorescein-labeled morpholino targeting the initiation of translation of the *SNAI2* mRNA, or a five base pair mismatch control morpholino, were electroporated into the epiblast of HH stage 3–4 embryos using a protocol in which only epiblast cells are targeted [2]. Embryos were incubated for 6–16 hours to allow cells in control embryos to undergo EMT and migrate into the mesoderm. Embryos were then processed for immunofluorescence to identify morpholino containing cells and Slug protein.

**Fig 6:**
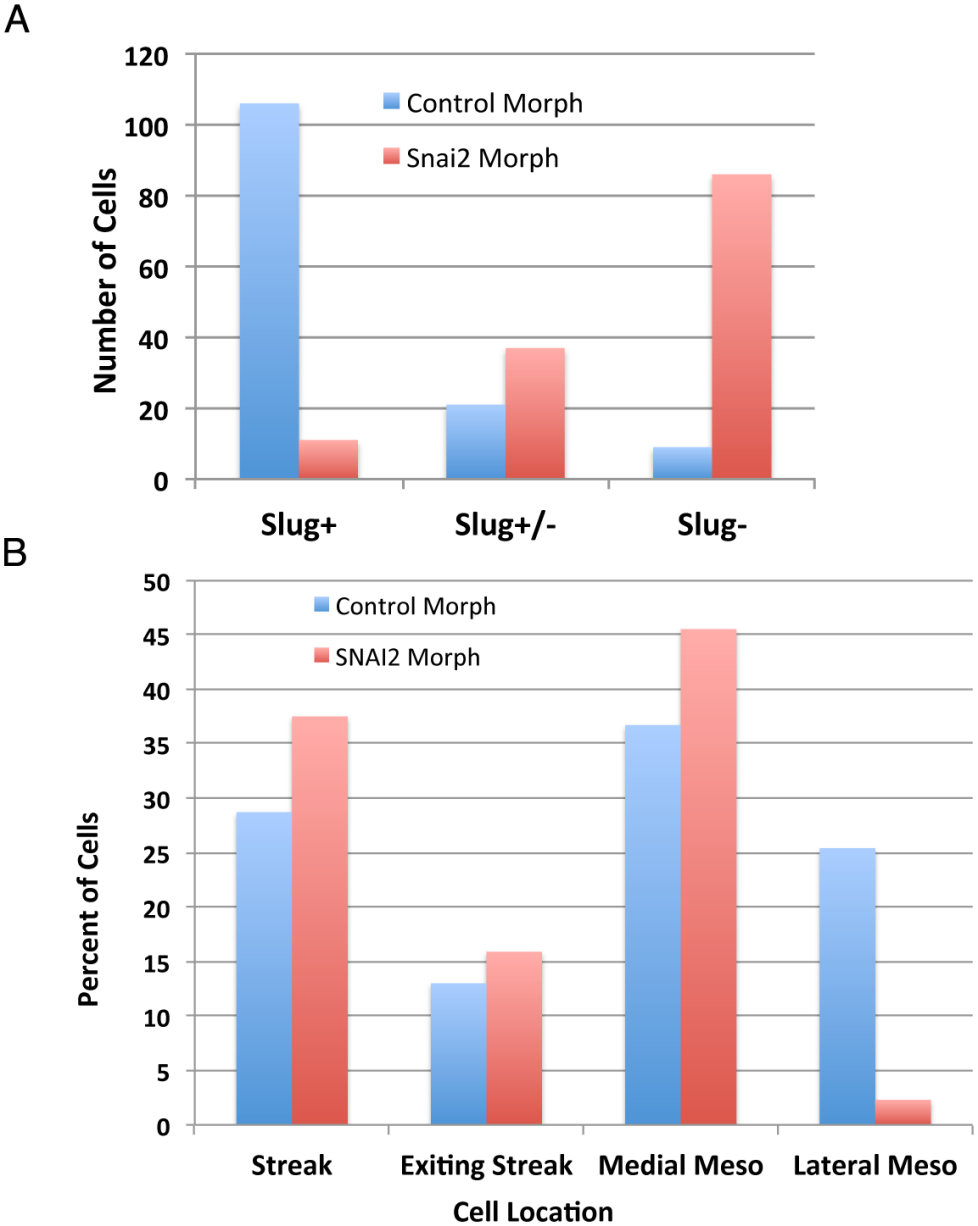
Effects of SNAI2 morpholino on Slug protein expression and cell location. (A) Reduction of Slug protein in most cells electroporated with morpholino targeting the initiation of translation of the Slug (Snail2) mRNA compared to the control (five base pair mismatch) morpholino. While most cells containing the control morpholino show high levels of Slug protein eight hours following electroporation as assayed by immunofluorescence, almost all cells containing the SNAI2 morpholino showed reduced or undetectable levels of Slug. (B) Relative location of epiblast cells electroporated with control or SNAI2 morpholino eight hours following electroporation, and the percent of cells at each location that are Slug-positive. Numbers of cells are shown above each bar. Whereas the percent of cells containing control morpholinos that are Slug-positive is similar at all locations, a higher percentage of Slug-positive cells containing the SNAI2 morpholinos were observed in the mesoderm away from the streak, indicating that SNAI2 morpholino containing cells lacking detectable Slug expression were preferentially located near the primitive streak.

**Fig 7:**
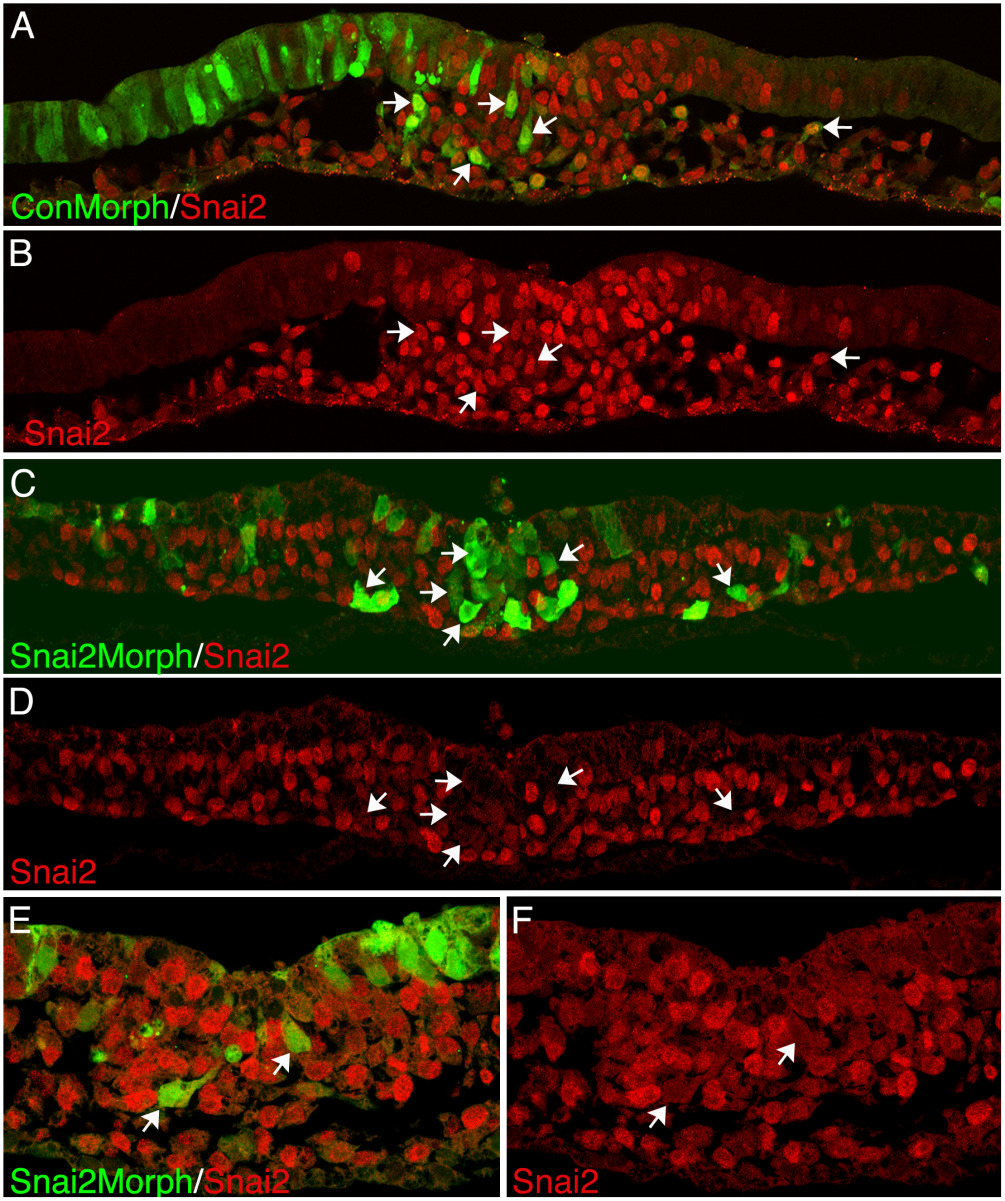
Location of SNAI2 morpholino and control morpholino containing cells in transverse sections of embryos. A fluorescein-labeled morpholino targeting the initiation of translation of the *SNAI2* mRNA, or a five base pair mismatch control morpholino, were electroporated into the epiblast of HH stage 3–4 embryos. (A-B) The same microscopic field visualizing cells containing the control morpholino (green) and Slug (red) in (A), or Slug (B). Almost all cells containing the control morpholino also express Slug protein (white arrows). (C-D) The same microscopic field visualizing cells containing the SNAI2 morpholino and Slug (C), or Slug (D). Most cells in the primitive streak containing SNAI2 morpholinos were Slug negative (white arrows). (E,F) Higher magnification views showing cells containing the SNAI2 morpholino, and lacking detectable Slug protein, undergoing EMT (arrows).

Almost all cells containing the *SNAI2* morpholino within the normal domain of Slug expression showed reduced or undetectable Slug protein levels (Figs 6A and 7C–7F). Morpholino-positive/Slug-negative cells were observed at all levels of the primitive streak and in the mesoderm (Fig 7C–7F), including bottle shaped cells undergoing EMT (arrows in Fig 7E and 7F) and rounded cells in the ventral streak (arrows in Fig 7C). *SNAI2* morpholino-positive/Slug protein negative cells were also observed throughout the mesoderm. However, the proportion of *SNAI2* morpholino containing cells lacking detectable Slug protein was reduced in the medial, mid and lateral mesoderm compartments relative to cells containing control morpholino (Fig 6B), suggesting a negative effect on cell migration. Since immunofluorescence is relatively insensitive, some morpholino containing cells lacking detectable Slug could retain functional protein levels. In chick, SNAI1 is not normally expressed in the primitive streak (see SNAI1 entry in the GEISHA database, http://geisha.arizona.edu). SNAI1 transcripts were not detected in cells containing SNAI2 morpholino (data not shown). Zeb1 and 2 are expressed in the avian primitive streak (http://geisha.arizona.edu), and so it is possible that in the absence of Slug protein (*SNAI2*), Zeb1 and/or Twist2 could function redundantly to enable EMT.

### Snail expression is insufficient to induce EMT in epiblast cells

Published studies have reported conflicting results regarding the ability of the Snail transcription factors to induce EMT in gastrula stage chicken epiblast [2, 26]. To more fully investigate the relationship between Snail protein expression and EMT in the epiblast, the effect of Snail overexpression in the epiblast was investigated using plasmids expressing a degradation resistant form of human *SNAI1* (6SAhSNAI1) that shows strong nuclear localization and an enhanced ability to induce EMT in a variety of cell types [22], or wild type chicken SNAI2 (WTcSNAI2). Expression constructs were electroporated into the lateral epiblast of pregastrula through HH stage 4 embryos. An expression construct containing the Rho inhibitor peptide C3 was used as a positive control for EMT induction. Although C3 expression has been shown to inhibit EMT in the neural crest[27], previous published findings clearly show that C3 expression induces EMT in the epiblast [1]. Multiple embryos were electroporated with each construct and fixed for processing at 4, 8 or 16 hrs.

**Fig 8:**
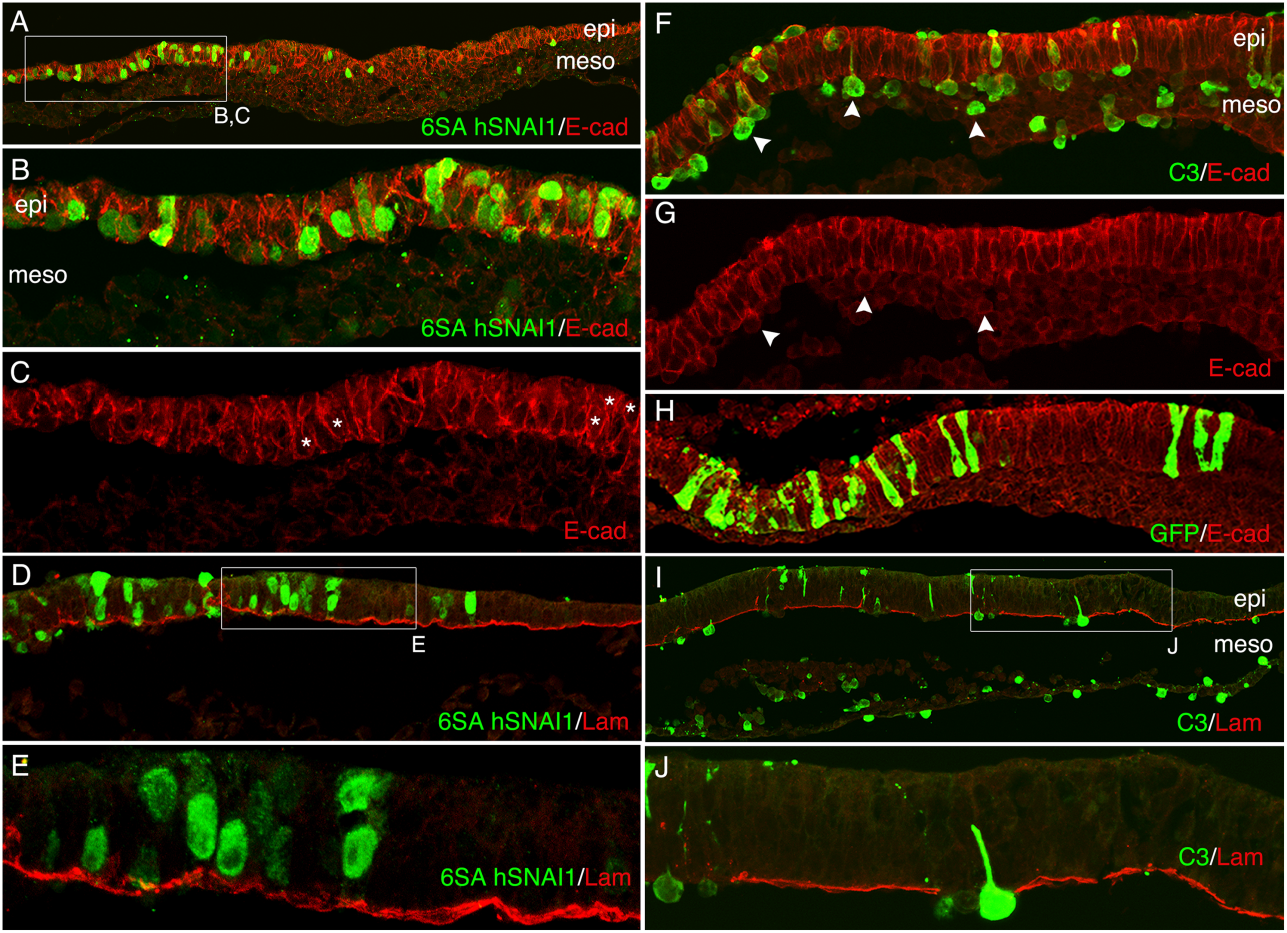
SNAIL over expression is insufficient to induct EMT in the epiblast. Transverse sections of embryos following electroporation of the epiblast with plasmids expressing a FLAG-tagged degradation resistant form of human Snail (6SAhSNAI1; A-E), myc tagged Rho-inhibitor peptide C3 (F, G, I, J) or pBEGFP (H). (A-C) Eight hours following electroporation with 6SAhSNAI1, no 6SAhSNAI1 expressing cells were observed in the mesoderm or exiting the epiblast. 6SAhSNAI1-expressing epiblast cells showed levels of E-cad and/or P-cad similar to untransfected cells. (D, E) Expression of 6SAhSNAI1 failed to induce basal lamina breakdown. (F,G) The same microscopic showing the epiblast and mesoderm of an embryo 4 hours following electroporation of the epiblast with the C3 expression construct. Virtually all C3-expressing cells (green) are observed in the mesoderm or exiting the epiblast (arrows). Some C3 positive cells were observed above the epiblast. (H) All cells expressing GFP remained in the epiblast. (I, J) Expression of C3 in epiblast cells induced basal lamina breakdown.

**Fig 9:**
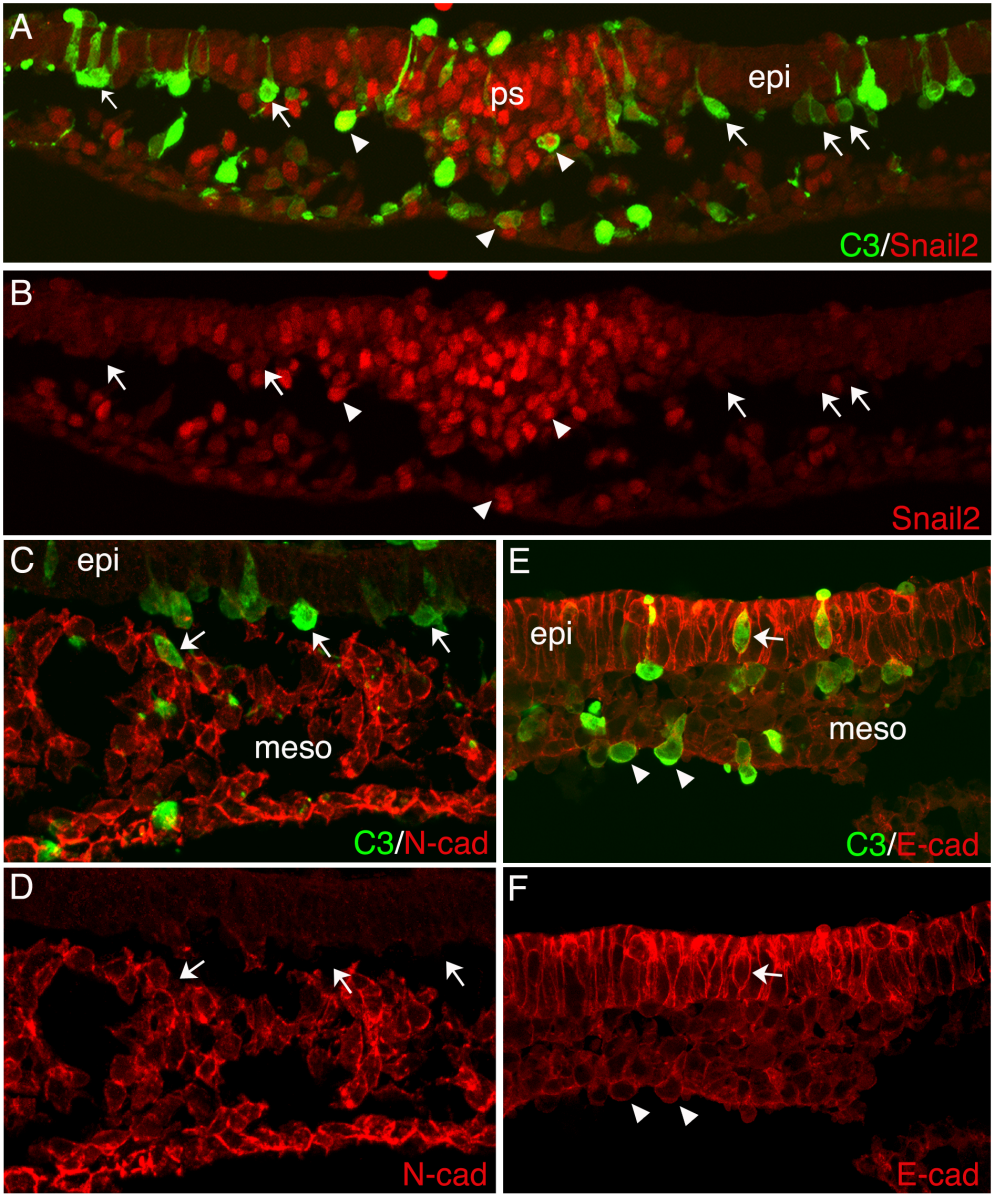
C3 induces EMT without activating Slug or upregulating N-cad. (A,B) Transverse sections showing the same microscopic field four hours following electroporation with the C3 expression vector. While cells expressing C3 (green) in the primitive streak region within the domain of normal Slug expression were also Slug-positive (arrowheads in A, B), C3 expressing cells in more lateral regions that were exiting the epiblast or that had migrated into the mesoderm failed to express detectable levels of Slug (arrows in A, B). (C, D) C3 expressing cells that had undergone EMT and migrated into the mesoderm failed to express N-cad.

Regardless of embryo stage at the time of electroporation or the duration of the experiment, cells expressing 6SAhSNAI1 remained within the epiblast (Fig 8A–8E, Table 1). Although 6SAhSNAI1 expression led to the downregulation of E-cad mRNA levels in the epiblast (S1 Fig), electroporated epiblast cells retained E-cad and/or P-cad protein at levels that appeared indistinguishable from control epiblast cells (Fig 8A–8C; [2]). Identical results were obtained using the WTcSnail2 expression vector (Table 1). In contrast, C3 expression induced rapid EMT, with the majority of electroporated cells having exited the epiblast within four hours following electroporation (Fig 8F and 8G). C3 positive cells that had migrated into the mesoderm showed reduced but detectable levels of E-cad/P-cad similar to the levels observed in adjacent lateral mesodermal cells. As previously reported [1], the basal lamina immediately beneath C3 expressing cells was lost, a prerequisite for cells to undergo EMT (Fig 8I and 8J). 6SAhSNAI1 expression failed to induce basal lamina breakdown (Fig 8D and 8E). C3 expressing cells in the lateral epiblast that were undergoing EMT or that had migrated into the mesoderm failed to upregulate Slug (Fig 9A and 9B) or N-cad (Fig 9C and 9D). Together, these results show that Snail expression is unable to induce EMT in the epiblast of early gastrula through late gastrula embryos, while Rho inhibition can rapidly induce EMT in the absence of Slug expression.

**Table 1:** EMT Induction by Snail and C3.

*Construct*	*GFP*		*cSNAI2*	*6SAhSNAI1*		*C3*	
Cell Location	*8hr*	*16hr*	*16hr*	*8hr*	*16hr*	*4hr*	*16hr*
in epiblast	526	107	56	347	98	546	0
exiting epiblast	0	0	0	0	0	38	1
in mesoderm	0	3	1	2	0	38	76
above epiblast	0	0	0	0	0	387	40

## Discussion

Cadherin switching is observed in many tissues and cell layers during embryonic development, where it can drive cell shape changes that are critical for morphogenesis [28]. Cadherin switching is also associated with certain human diseases, including cancer metastasis [29, 30]. During gastrulation, an evolutionarily conserved cadherin switch occurs in which E-cad expressed in the epiblast is downregulated as cells undergo EMT, while concomitantly N-cad expression is upregulated in emerging mesoderm and endoderm cells. In this study, we have examined the relationship between the expression of the two closely related epithelial cadherin genes *CDH1* (E-cad) and *CDH*3 (P-cad), EMT, and Slug function during chick gastrulation. We find that P-cad is present at robust levels on the surface of cells in the epiblast, primitive streak and early mesoderm, diminishing only once mesoderm cells have migrated away from the streak. Although antibodies that can specifically recognize E-cad are not available, E-cad mRNAs are present in the epiblast but not in the primitive streak or mesoderm. Assuming that E-cad protein distribution roughly corresponds to its mRNA distribution, these findings are consistent with the possibility that E-cad protein is diminished or absent in the primitive streak and mesoderm, while P-cad protein persists as cells undergo EMT and emerge into the mesoderm. The persistence of E-cad and/or P-cad around the periphery of newly formed mesodermal cells is also observed in mouse embryos (Fig 3D and 3E; [31]). We also find that overexpression of E-cad in individual primitive streak cells fails to inhibit EMT. Similar findings have been reported in *Drosophila* [16], and a recent study has shown that in mammary epithelial cells Twist-induced EMT requires E-cad [17].

### The role of Slug during avian gastrulation

The transcription factor Slug (*SNAI2*) was first identified in avian embryos as a regulator of cell behavior during gastrulation and neural crest formation [32]. Many subsequent studies have shown that Slug and the closely related protein Snail (*SNAI1*) directly repress transcription of E-cad (*CDH1*) and other genes involved in maintaining the epithelial phenotype. A model therefore arose in which EMT was triggered by the transcriptional activation of *SNAI1* and/or *SNAI2*, leading to the loss of E-cad followed by the onset of EMT. Additional transcriptional repressors, including Twist and Zeb, have been shown to combine with Snail proteins to repress the epithelial phenotype. In the *SNAI1* knockout mouse, (*SNAI1* is expressed in the primitive streak of mammals while SNAI2 is expressed in the chicken primitive streak), epiblast cells undergo gastrulation to form a mesoderm cell layer, though the mesoderm cells retain some epithelial characteristics including a polarized phenotype and intercellular junctions [33], identifying a direct or indirect role for Snail in the downregulation of the epithelial phenotype. Here we reexamined the role of Slug in EMT regulation using a morpholino targeting the initiation of translation of the *SNAI2* mRNA. Although Slug protein levels were undetectable in most morpholino-containing cells, the movement of cells from the epiblast to the mesoderm through the primitive streak was not inhibited. Mesoderm cells lacking detectable Slug protein showed reduced ability to migrate into the lateral mesoderm, though morphologically they appeared indistinguishable from control cells.

As another approach to investigating the contribution of Slug to EMT regulation, chicken Slug or a degradation resistant form of human *Snail1* were expressed in epiblast cells. Previous studies have reported conflicting results regarding the ability of Slug/Snail to induce EMT in the epiblast [2, 26]. Regardless of the stage at which embryos were electroporated (HH stage 1–6), the location of electroporated cells, or the duration of the experiment (4–16 hours), in no case did we observe cells expressing chicken Slug or the degradation resistant human *Snail1* undergoing EMT or residing in the underlying mesoderm. Conversely, as previously reported [1] expression of the Rho inhibitor peptide C3 rapidly induced EMT in epiblast cells. C3 expressing epiblast cells were observed undergoing EMT almost as soon as the protein could be detected, indicating a rapid response to Rho-inhibition. C3-induced EMT is independent of transcriptional activation of Slug that is normally upregulated prior to the onset of EMT in the primitive streak (Fig 1G and 1H) or N-cad that is normally upregulated in emerging mesoderm cells (Fig 4). During gastrulation in control embryos, it is likely that inhibition of Rho activity occurs in response to a transcriptional regulatory pathway activated in the primitive streak [2]. Nevertheless it is informative that Rho inhibition alone can rapidly and directly induce the cell biological aspects of EMT.

The results presented here provide new information concerning the relationship between cadherin switching and EMT during gastrulation. The finding that E-cad and P-cad mRNAs, and likely also the proteins, are differentially expressed raises questions about their respective functions during gastrulation. While E-cad is apparently downregulated prior to the onset of EMT, P-cad is present on the surface of cells in the epiblast, primitive streak and newly formed mesoderm. Although the intracellular domains of E-cad and P-cad are identical, the extracellular domains have diverged and so they may have evolved differential adhesion properties and functions that are important for EMT. The striking re localization of epithelial cadherins from lateral cell surfaces in epiblast cells to circumferential in the primitive streak may lead to reduced intercellular adhesion that might also facilitate EMT. In this scenario, the loss of adherens junctions apically and interactions with basement membrane basally would allow epithelial cadherins (and other cell surface proteins) to redistribute to the entire cell surface, reducing intercellular adhesion. Although mechanisms regulating the loss of adherens junctions are not fully defined, Nakaya et al[1] have shown that the downregulation of the RhoGEF Net1 as epiblast cells approach the primitive streak leads to loss of basally localized RhoA activity and dissolution of the basement membrane [1]. The upregulation of N-cad and presumptive loss of E-cad may further reduce intercellular adhesion. These possibilities remain to be tested.

## Supporting Information

S1 Fig Expression of 6SAhSNAI1 in the epiblast led to down regulation of E-cad mRNA. (A, B) The same microscopic field showing lateral epiblast cells expressing GFP (green) and E-cad mRNA (purple). (C, D) Epiblast cells expressing C3 had migrated into the mesoderm and had downregulated E-cad mRNAs. (E,F) Epiblast cells expressing 6SAhSNAI1 remained in the epiblast (F) but showed greatly reduced levels of E-cad mRNAs (brackets in E).
(TIF)
