# Supplementary figures and images for: Gastrulation EMT Is Independent of P-Cadherin Downregulation
Source: PLoS One. 2016 Apr 20;11(4):e0153591. doi: 10.1371/journal.pone.0153591 (PMC4838233; doi:10.1371/journal.pone.0153591)

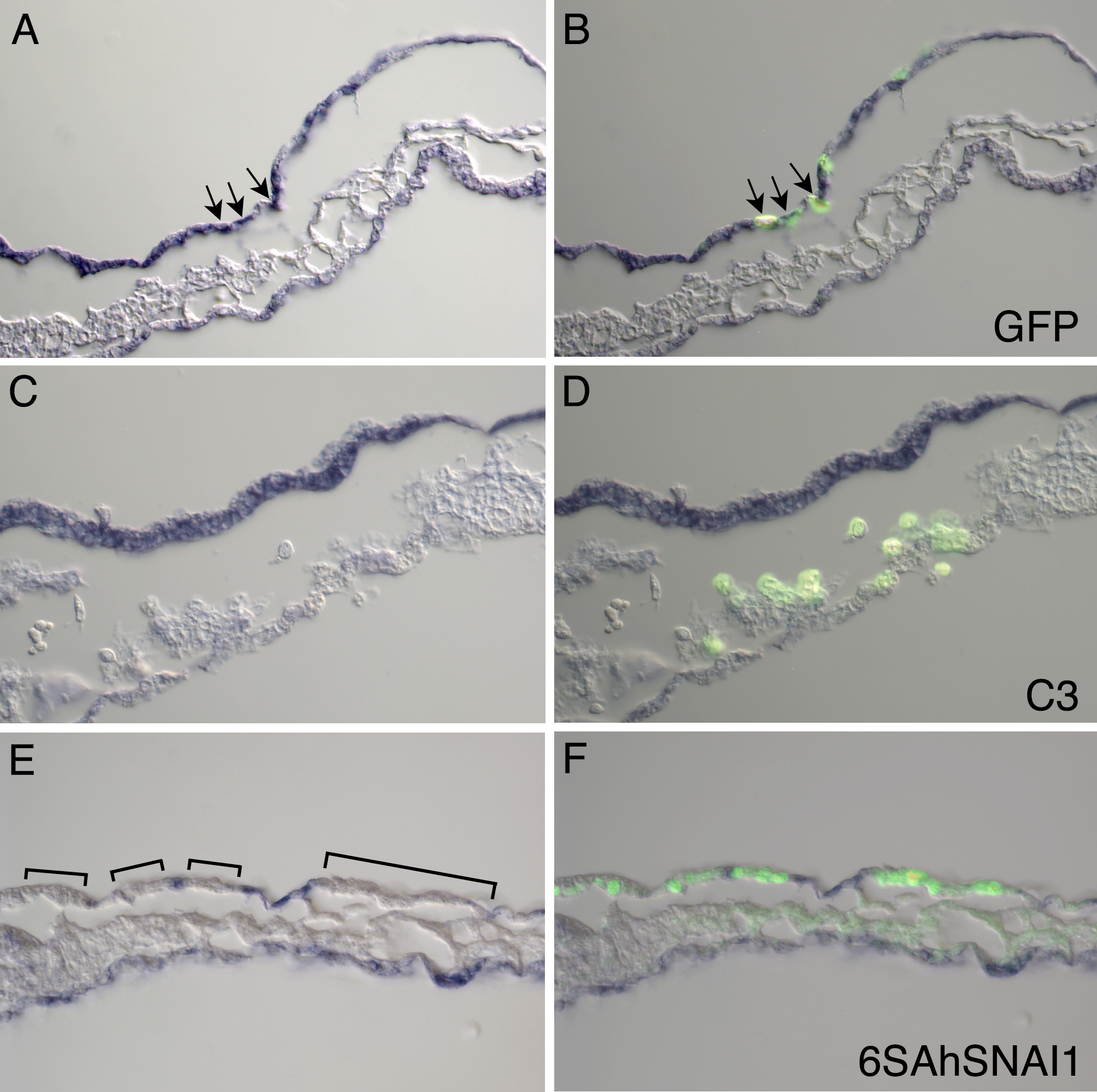

Supplement: S1 Fig — (A, B) The same microscopic field showing lateral epiblast cells expressing GFP (green) and E-cad mRNA (purple). (C, D) Epiblast cells expressing C3 had migrated into the mesoderm and had downregulated E-cad mRNAs. (E,F) Epiblast cells expressing 6SAhSNAI1 remained in the epiblast (F) but showed greatly reduced levels of E-cad mRNAs (brackets in E). (TIF) [file pone.0153591.s001.tif]
